# Assessment of common mental disorders in the Avon Longitudinal Study of Parents and Children (ALSPAC) birth cohort at age 24 years using the Clinical Interview Schedule-Revised (CIS-R)

**DOI:** 10.12688/wellcomeopenres.23933.1

**Published:** 2025-12-22

**Authors:** Sophie J Fairweather, Holly Fraser, Nicholas A Donnelly, Hannah J Jones, Rebecca M Pearson, Glyn Lewis, Golam M Khandaker

**Affiliations:** 1 MRC Integrative Epidemiology Unit, Bristol, England, UK; 2 NIHR Bristol Biomedical Research Centre, Bristol, England, UK; 3 Avon and Wiltshire Mental Health Partnership NHS Trust, Bath, England, UK; 4 Centre for Academic Mental Health, University of Bristol Medical School, Bristol, England, UK; 5 Manchester Metropolitan University Department of Psychology, Manchester, England, UK; 6 University College London Division of Psychiatry, London, England, UK

**Keywords:** Common Mental Disorders, Depression; Anxiety; CIS-R; Population Health; Symptom; ALSPAC; Longitudinal; Clinical Interview

## Abstract

Common mental disorders (CMDs), such as depression and anxiety, are highly prevalent and often manifest first between childhood and early adulthood. These disorders in young people can have wide-reaching negative impacts on health and other domains throughout the life course. Therefore, studies of CMDs in young people are essential to understand their prevalence, risk factors, and prognosis. In addition to diagnoses, studies of the individual symptom dimensions of depression and anxiety are required to understand the complex and heterogeneous presentation of CMDs.

Questionnaires frequently used to assess depression and anxiety in large-scale population-based studies, though useful, are often unable to provide validated categorical diagnoses. The Clinical Interview Schedule-Revised (CIS-R) is a validated, internationally recognised, standardised semi-structured interview tool for assessing CMD symptoms in large community-based samples. It provides information on individual symptoms, symptom dimensions, and ICD-10 diagnoses for a range of CMDs.

This Data Note describes CIS-R data collected from a prospective birth cohort in Southwest England, the Avon Longitudinal Study of Parents and Children (ALSPAC), at age 24 years. We summarise the available data and present them in hierarchical order from International Classification of Diseases (ICD)-10 diagnosis, symptom dimension scores, individual symptom scores, and symptom directionality. We also present the descriptive characteristics of the data. For example, we report the prevalence of ICD-10 depression (11%) and anxiety disorders (10%) and report five symptom domain scores (somatic, psychological, atypical, anxiety, and depressive cognitions) in addition to many individual symptoms. The aim of this Data Note is to enhance the visibility of available data at age 24 in ALSPAC, document data generation and coding processes, and provide information about the derived variables for use by the wider scientific community.

## Introduction

Common mental disorders (CMDs) is an umbrella term that refers to the most common mood, emotion, or affect-related mental health conditions ^1^. In this group, anxiety and depression are the most prevalent, with approximately 581 million people worldwide estimated to be living with either disorder in 2019 ^1^. Other CMDs include panic disorder, phobias, obsessive-compulsive disorder (OCD) and post-traumatic stress disorder (PTSD). Psychotic disorders such as schizophrenia and bipolar affective disorder are not included in the CMD category.

It is widely understood that many CMDs often first manifest in adolescence ^2^, with the transitional period between adolescence and young adulthood emphasised as a time of unique vulnerability to mental health challenges. Evidence suggests that the burden of CMDs in young people has been increasing, with data showing a sharp increase in the rates of mental illness in young people since the early 2000s ^3^. Incidence rates in young people during this period have doubled ^4^, with the need for service provision for affective disorders in adolescence reflecting this increase ^5^. This underscores the importance of high-quality measures of CMD symptoms and prevalence in the population to define and characterise the presentation of adverse mental health outcomes.

Understanding the prevalence of CMDs is important for general health and well-being. Depression and anxiety often lead to significant deterioration in quality of life ^6^, a higher risk of physical health conditions ^7^, impaired social functioning ^8^ and relational issues with partners ^9^ and peers ^10, 11^. CMDs are also associated with poorer access to care ^12^ and increased sick leave from work ^13^, and are a leading risk factor for suicide ^14^.

High prevalence of depression in young people is of particular concern, with depression increasing the risk of multiple negative functional outcomes in later life. These include anxiety, impaired social functioning, and a higher vulnerability to criminality ^15^. Comorbid depression and anxiety is an additional risk factor for increased adverse outcomes in early adulthood ^16^. Depression and anxiety in this age group are also associated with NEET (“Not in education, employment or training”) status in young people ^17^.

To understand the life course trajectories of young people with mood and affective disorders, rich longitudinal data are required. The Avon Longitudinal Study of Parents and Children (ALSPAC) birth cohort contains intergenerational data on a range of biological, psychological, and social characteristics from birth to adulthood. Therefore, this cohort is well-positioned to support research questions related to the aetiology and characteristics of mental health conditions. ALSPAC data have been used extensively to examine an array of mental health conditions and their characteristics, including the complex determinants of depression ^18– 25^ and anxiety ^26– 28^ as well as other psychiatric conditions like schizophrenia ^29, 30^, and psychosis ^31– 34^.

In large scale population-based studies, depression and anxiety have primarily been assessed using measurement scales and instruments that assess the prevalence of symptoms. These are often delivered in a questionnaire format and are used to generate dimensional and symptom level scores. Alternative methods include structured and semi-structured interviews, which offer more granularity when assessing symptoms through branching architecture that facilitates the measurement of frequency and severity of symptoms in a standardized format ^35^.

The Clinical Interview Schedule-Revised (CIS-R) is an internationally recognised, validated, and standardised semi-structured interview tool for assessing CMD symptoms in large community-based samples ^36^. It can provide information on individual symptoms, symptom dimensions, and ICD-10 diagnoses of a range of CMDs.

The aim of this data note is to describe the characteristics of the CIS-R data collected from ALSPAC participants aged 24 years. We describe the ALSPAC study, including how to request and access data. We comprehensively describe the collection and format of CIS-R data within the ALSPAC resource. We detail the derivation processes relating to the extraction of both symptom and diagnosis level data within the dataset to facilitate reproducible analyses. We then summarise the available data, describe their characteristics, and highlight the demographic characteristics of the clinical population. We also discuss the potential limitations of the data and key considerations for use. Historically, ALSPAC data users have combined CIS-R items in different ways for bespoke use; an additional aim of this data note is to encourage the standardisation and consistency of data use.

## Materials and methods

### ALSPAC cohort description

The ALSPAC birth cohort collected data from pregnant women residing in Avon, UK, with expected delivery dates between April 1991 and 31st December 1992 ^37, 38^. The initial number of pregnancies enrolled in the study was 14,541. Of the initial pregnancies, 13,988 children were alive at 1 year of age (G1 children) ^39^. When the oldest children were approximately seven years of age, an attempt was made to bolster the initial sample with eligible cases who had not joined the study originally. As a result, the total ALSPAC sample size after seven years of age was 15,447 pregnancies, resulting in 15,658 foetuses. Of these, 14,901 children were alive at 1 year of age. Of the original 14,541 initial pregnancies, 338 were from women who had already enrolled with a previous pregnancy, meaning that 14,203 unique mothers were initially enrolled in the study. As a result of the additional phases of recruitment, a further 630 women who did not enrol originally have provided data since their child was 7 years of age. This provides a total of 14,833 unique women (G0 mothers) enrolled in ALSPAC as of September 2021.

Study data have been collected using both paper and online methods and are managed using Research Electronic Data Capture (REDCap) ^40, 41^ tools hosted at the University of Bristol. REDCap is a secure web-based software platform designed to support data capture for research studies.

The study website contains details of all available data through a fully searchable data dictionary and variable search tool ( http://www.bristol.ac.uk/alspac/researchers/our-data/). Ethical approval for the study was obtained from the ALSPAC Ethics and Law Committee and the Local Research Ethics Committees.

### Mental health measures available in ALSPAC

**Figure 1.**
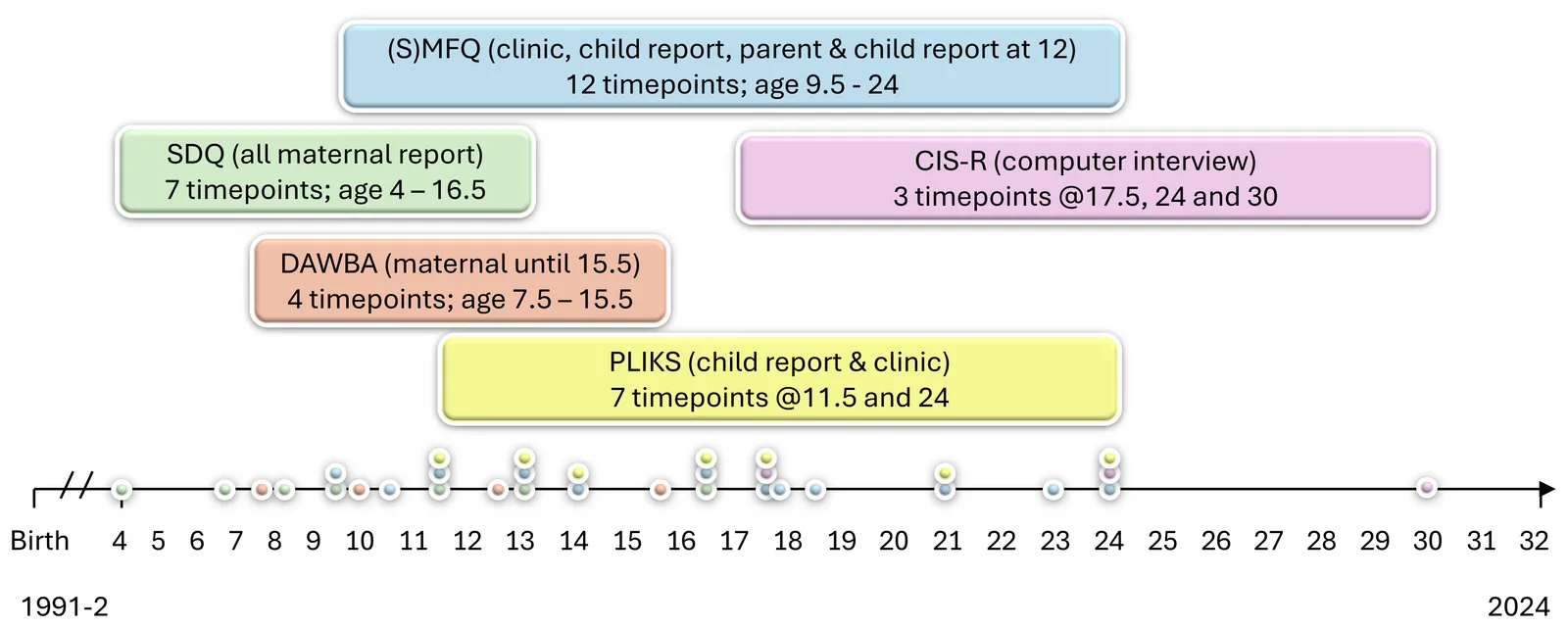
Timeline of all G1 mental health measure data available in ALSPAC from ages 4 to 24 (approximate) ^1^.

The ALSPAC study has repeatedly collected data using a range of mental health screening tools, including the Strengths and Difficulties Questionnaire (SDQ) (measured at seven time points between ages 4 and 16), the Development and Wellbeing Assessment (DAWBA) (measured at four time points between age 7/8 and age 13), the Short Mood and Feelings Questionnaire (SMFQ) (measured at ten timepoints between ages 10 and 28), and the Clinical Interview Schedule-Revised (CIS-R) at ages 18, 24, and 30. Figure 1 shows a timeline schematic of the key mental health measures available for G1 offspring in ALSPAC up to the age of 24.

### The Revised Clinical Interview Schedule (CIS-R)

The Clinical Interview Schedule (CIS) was developed to facilitate the assessment of CMDs in community settings, with the aim of standardising measurements of individual symptoms of illness and aiding the diagnostic assessment of CMDs ^42^. The Revised CIS (CIS-R) was later developed to improve and further standardise assessment when measuring CMDs using the CIS ^36^ and to increase suitability for administration by non-clinical interviewers in community, occupational, and primary care research ^36^. It has been shown to be highly sensitive when detecting CMDs in the population compared to electronic primary care data ^43^ and can be used to assign ICD-10 diagnostic categories ^44^ as well as measure individual symptoms.

The CIS and CIS-R have been used in a variety of research domains, including, for example, research exploring the relationship between depression and sedentary behaviour in adolescents ^45^, alcohol dependence and depression in adolescents ^46^, the relationship between depression and socioeconomic factors ^47^ such as housing and neighbourhood quality ^48^, and in retrospective form to assess depression relapse in primary care patients receiving antidepressant treatment ^49^.

The CIS-R has been used to measure the prevalence of treated and untreated psychiatric disorders in the Adult Psychiatric Morbidity Survey (APMS), a large-scale population study conducted every seven years in England and Wales ^50^, and in many randomised controlled trials (RCTs) ^47^. It has been used successfully in different global populations ^51, 52^ and in older populations, with a sensitivity of 75% and specificity of 98% for detecting depressive episodes in people over the age of 60 ^53^.

### CIS-R assessment of ALSPAC cohort participants at age 24y

The computerized version of the CIS-R was self-administered by cohort participants in face-to-face assessment clinics at age 24 years. The computerized version of the interview shows close agreement with the interviewer administered version ^54, 55^ and has been used to derive diagnoses for depression, generalised anxiety disorder (GAD), phobia, and panic disorder according to ICD-10 criteria. The CIS-R also includes a preliminary section on physical health symptoms that is not described here. After this initial section, which asks questions on respondents’ overall physical health and wellbeing, the questionnaire progresses to 14 sections. Each section assesses a different symptom occurring in the last two weeks, and responses can be summed to give a 0–4 or 0–5 score. A shortened version of the CIS-R was used at age 24 years in ALSPAC, which did not include items on obsessions, compulsions, and worries about physical health.

Sections:

1. Somatic symptoms (aches/pains)
2. Fatigue
3. Concentration and forgetfulness
4. Sleep problems
5. Irritability
6. *Worry about physical health [not included at age 24 ALSPAC clinic]*
7. Depression (low mood and anhedonia)
8. Depressive ideas
9. Worry
10. Anxiety
11. Phobias
12. Panic
13. *Obsessions [not included at age 24 ALSPAC clinic]*
14. *Compulsions [not included at age 24 ALSPAC clinic]*

There are additional differences between the interview sections of the CIS-R presented here and those of the original CIS-R ^36^. Please see the ‘considerations for the data’ section for a detailed description of these differences.

### Derivation of age 24 CIS-R variables in ALSPAC

**Figure 2.**
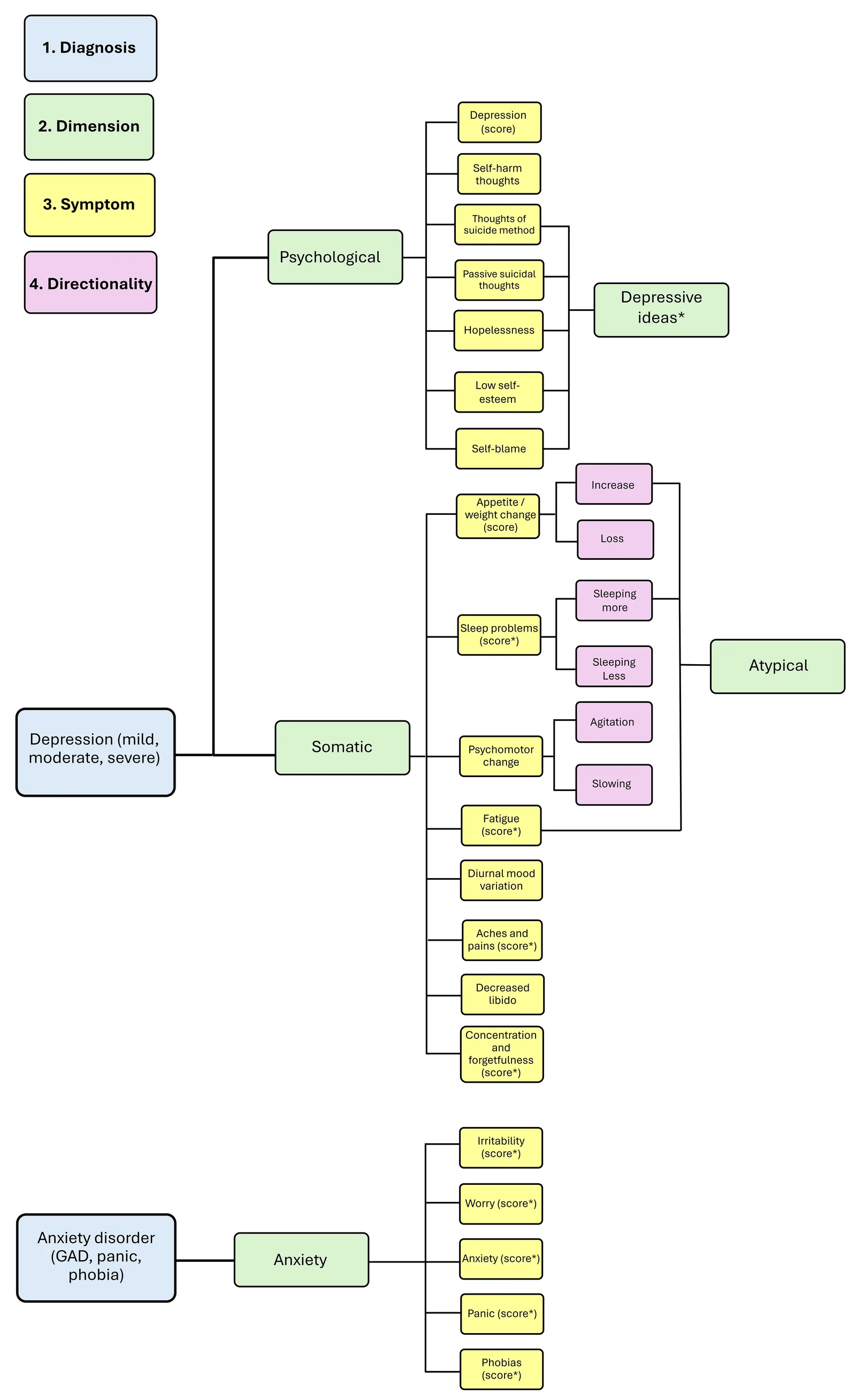
Schematic depiction of each level of CIS-R data, from diagnosis (level 1) to symptom directionality (level 4). Asterisks denote original CIS-R items.

There is a range of CIS-R variables available in the ALSPAC dataset that were derived from the original questionnaire item variables. These reflect different levels of granularity with respect to symptoms of depression and anxiety (ranging from categorical diagnoses (least granular) to the directionality of specific symptoms (most granular). Table 1 shows the hierarchy of data that we have denoted in levels as follows, with Figure 2 depicting the data hierarchy visually:

- Level 1: ICD-10 diagnosis
- Level 2: Symptom dimensions
- Level 3: Individual symptom scores
- Level 4: Symptom directionality

**Table 1.** CIS-R derived depression and anxiety data from the ALSPAC birth cohort at age 24.

Symptom/concept		Variable name
Level 1: ICD-10 diagnosis		
**L1**	Depression (mild, moderate or severe)	FKDQ1000
	Moderate/Severe depression	FKDQ1010
	Severe depression	FKDQ1020
	Any anxiety disorder	ICD10_any_anx_disorder
	Generalized anxiety disorder (GAD)	FKDQ1030
	Social phobia	FKDQ1050
	Specific/isolated phobia	FKDQ1060
	Panic disorder	FKDQ1070
Level 2: Symptom dimensions		
**L2**	Somatic symptom dimension	somatic_CISR_dimension_24
	Psychological symptom dimension	psych_CISR_dimension_24
	Anxiety symptom dimension	anx_CISR_dimension_24
	Depressive ideas dimension	depidea_CISR_dimension_24
	Atypical depressive symptom dimension	atypical_dep_CISR_dimension_24
Level 3: Individual symptoms		
**L3**	Fatigue score	fatigue_CISR_symp_24
	Concentration and forgetfulness score	conc_CISR_symp_24
	Irritability score	irrit_CISR_symp_24
	Worry score	worry_CISR_symp_24
	Anxiety score	anx_CISR_symp_24
	Phobias score	phob_CISR_symp_24
	Panic score	panic_CISR_symp_24
	Depression score	dep_CISR_symp_24
	Aches and pains score	aches_pains_CISR_symp_24
	Sleep problems score	sleep_CISR_symp_24
	Psychomotor change	pmotor_CISR_symp_24
	Appetite/weight change	wtchange_CISR_symp_24
	Low self esteem	esteem_CISR_symp_24
	Hopelessness	hopeless_CISR_symp_24
	Decreased libido	libido_CISR_symp_24
	Self-blame	blame_CISR_symp_24
	Diurnal variation in mood	diurnal_CISR_symp_24
	Self-harm thoughts	sharm_CISR_symp_24
	Passive suicidal thoughts	pass_suicidal_CISR_symp_24
	Thoughts of suicide method	suicide_intent_CISR_symp_24
	Anhedonia	anhedonia_CISR_symp_24
Level 4: Symptom directionality		
**L4**	Sleeping less	sleep_less_CISR_symp_24
	Sleeping more	sleep_more_CISR_symp_24
	Psychomotor agitation	agitation_CISR_symp_24
	Psychomotor slowing	slow_CISR_symp_24
	Appetite increase/weight gain	wtgain_CISR_symp_24
	Appetite decrease/weight loss	wtloss_CISR_symp_24

***ICD-10 diagnosis (level 1).*** ICD-10 diagnoses derived from the CIS-R data at age 24 included GAD, depression, panic disorder, and phobia anxiety disorders (agoraphobia, social phobia, and specific phobia). ICD-10 diagnoses are generated algorithmically according to an established coding framework that sums up clinically relevant symptoms and features of mental health disorders ^56^. Here, we briefly describe this process. First, the interview responses for each symptom group (e.g., fatigue and sleep problems) were summed to create within-symptom group scores. Scores for symptoms that are relevant to each psychiatric disorder are then summed to create an overall combined symptom score indicative of the severity of psychiatric illness. Thresholds were then used to categorize scores into severity and combined with interview responses relating to duration of symptoms, whereby the presence of symptoms for a minimum of 2 weeks or more indicates the presence of a psychiatric disorder. See Supplementary Table 1 for further details on the ICD-10 diagnosis derivation formulas using the CIS-R data.

***Symptom dimensions (level 2).*** There are many recognized groupings (i.e., dimensions or domains) of mood and anxiety symptoms that are used for research and sometimes for clinical purposes. For instance, depressive symptoms are often considered to belong to two distinct dimensions: somatic and psychological ^57– 59^. Recently, Lamers *et al.* ^60^ introduced the atypical symptom dimension which comprises the specific somatic symptoms of depression, such as energy intake/expenditure imbalance, increased appetite, weight gain, fatigue, and sleeping more.

***Dimension scores (level 2).*** we computed five symptom dimension scores (somatic, psychological, depressive ideas, atypical, and anxiety symptom dimension scores). Each of these represents the sum of the relevant individual symptom scores. Before summing, the individual symptom scores were dichotomized to ensure that each symptom contributed equally (i.e., equal weight) to the dimension score. It is important to note that these dimensions are not mutually exclusive. For example, the somatic and atypical dimensions overlap and share appetite/weight increase, increased sleep, and fatigue symptoms. 

***Individual symptoms (level 3).*** Individual symptom items were derived using interview responses related to specific granular constructs, such as fatigue, concentration, forgetfulness, and hopelessness. Unlike the dimension variables, these items are distinct (they do not overlap) and can be treated as individual variables. Items at this level are a mixture of scores (ordinal), discrete/categorical variables, and binary variables (see Table 4a and Table 4b).

***Symptom directionality (level 4).*** The final level of CIS-R data we derived indicates directionality within some specific symptoms such as weight/appetite gain or loss, psychomotor agitation or slowing, and sleeping less or more. Symptom directionality variables have been derived for those symptoms wherein the Level 3 score is clinically relevant in either direction (see Table 5a and Table 5b). For example, a high ‘sleep problems’ score could indicate either sleeping less or sleeping more.

In the supplementary materials, we provide a detailed coding framework describing how all four levels were derived from these original variables (Supplementary Table 2) with sufficient information for these processes to be reproducible for other researchers. We also provide a list of core and original questionnaire variables (corresponding to the 12 questionnaire sections, Supplementary Table 3).

**Figure 3.**
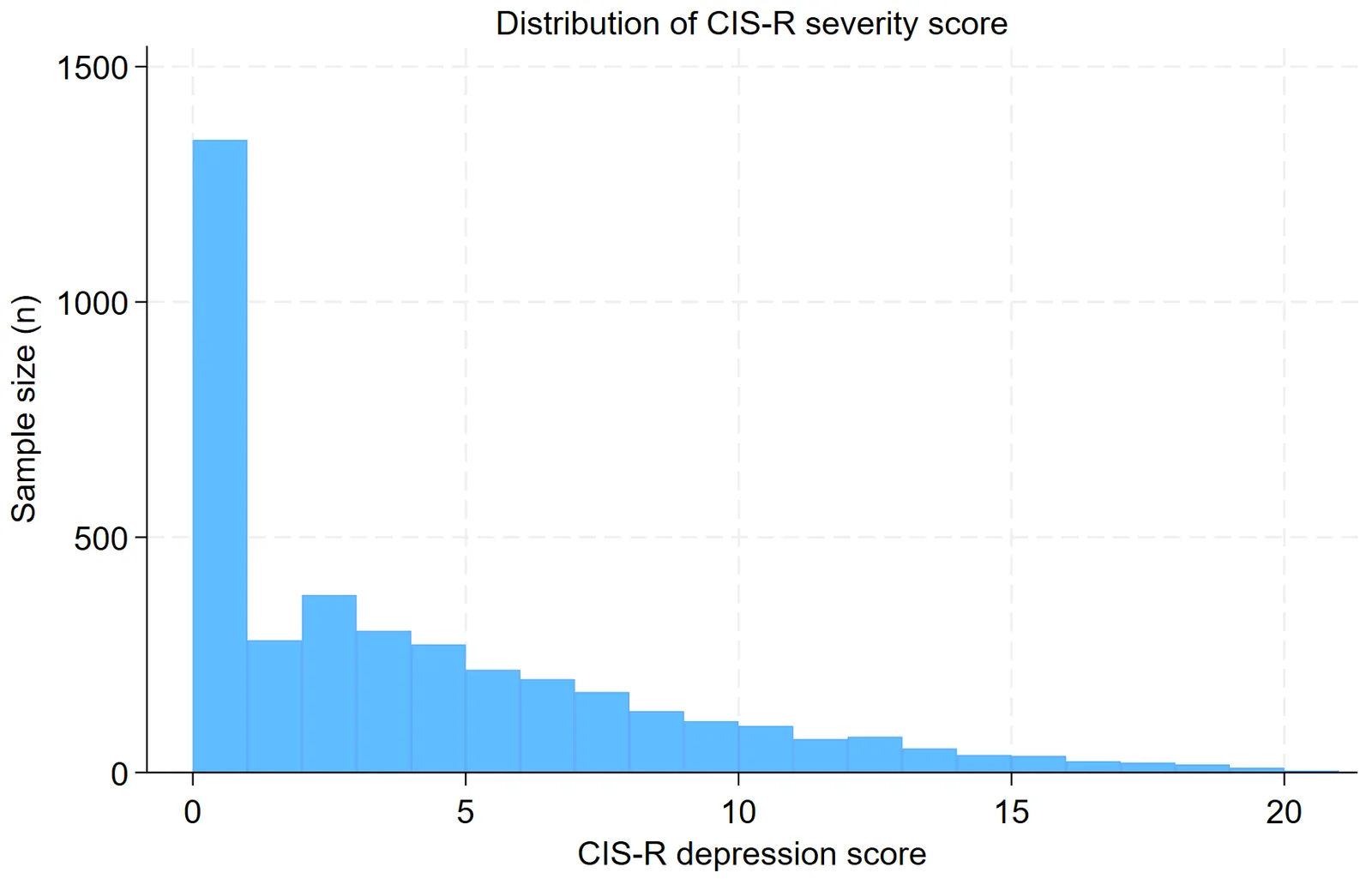
Histogram showing distribution of CIS-R depression severity score.

***Total symptom score.*** In addition to the variables in the four levels described above, we created a total depression symptom score. This is a continuous 0–21 score (see Figure 3), computed by summing the following individual symptom scores: depression, depressive ideas, fatigue, concentration and memory problems, and sleep problems ^61^.

***Assessment and coding.*** The ALSPAC CIS-R questionnaire sections are gated whereby all individuals are asked “mandatory” questions for each section. Those who respond “no” to these questions are not asked further questions in the section. This gating is simple in some instances, where the progression to further questions in the section is gated by a single question. However, in other instances, this is more complicated and there are several gating questions. We have indicated which questions are gated in Supplementary Table 3 and have provided a description of irregular gating as a footnote.

To make efficient use of the data, when using follow up questions to derive level 2, 3 and 4 data we include all initial “no” responders to the gating question as “no” responders; this assumes their response would have been no to all follow up questions in the section, taking a conservative approach. For example, if the participant says ‘no’ to having felt anxious or nervous in the past month they are not asked further questions regarding the nature of that anxiety, and are considered to be a ‘no’ on all further anxiety items. This is typical of an adaptive interview, with the CIS-R designed to emulate a real clinical encounter.

It should also be noted that in all instances where “or” logic was used to combine one or more variables, observations were included if at least one variable had data available, even if data were missing from other variables. 

Many of the derived variables are ordinal sum scores ranging from 0–4, or are created from dichotomised versions of the sum scores. It is important to note here that within the dichotomised variables, scores of zero do not indicate a complete absence of the symptom, as both absence and mild symptoms are included (for example, when coding the fatigue symptom score, young people with symptoms on four or more days in the past week scored 1, whereas those with symptoms between one and three days scored 0). This is because CIS-R applies a higher threshold while scoring (i.e., classifying a response as “yes” = symptom present) than commonly used questionnaires. Please see the Supplementary Information for further details. As such, the data were coded in this way to differentiate respondents whose symptoms met clinical significance from those with a lower symptom severity who may not meet the threshold of clinical significance, for example, reporting the presence of a symptom for <=4 days within the past week.

## Results

### Characteristics of data generated using the CIS-R

**Figure 4.**
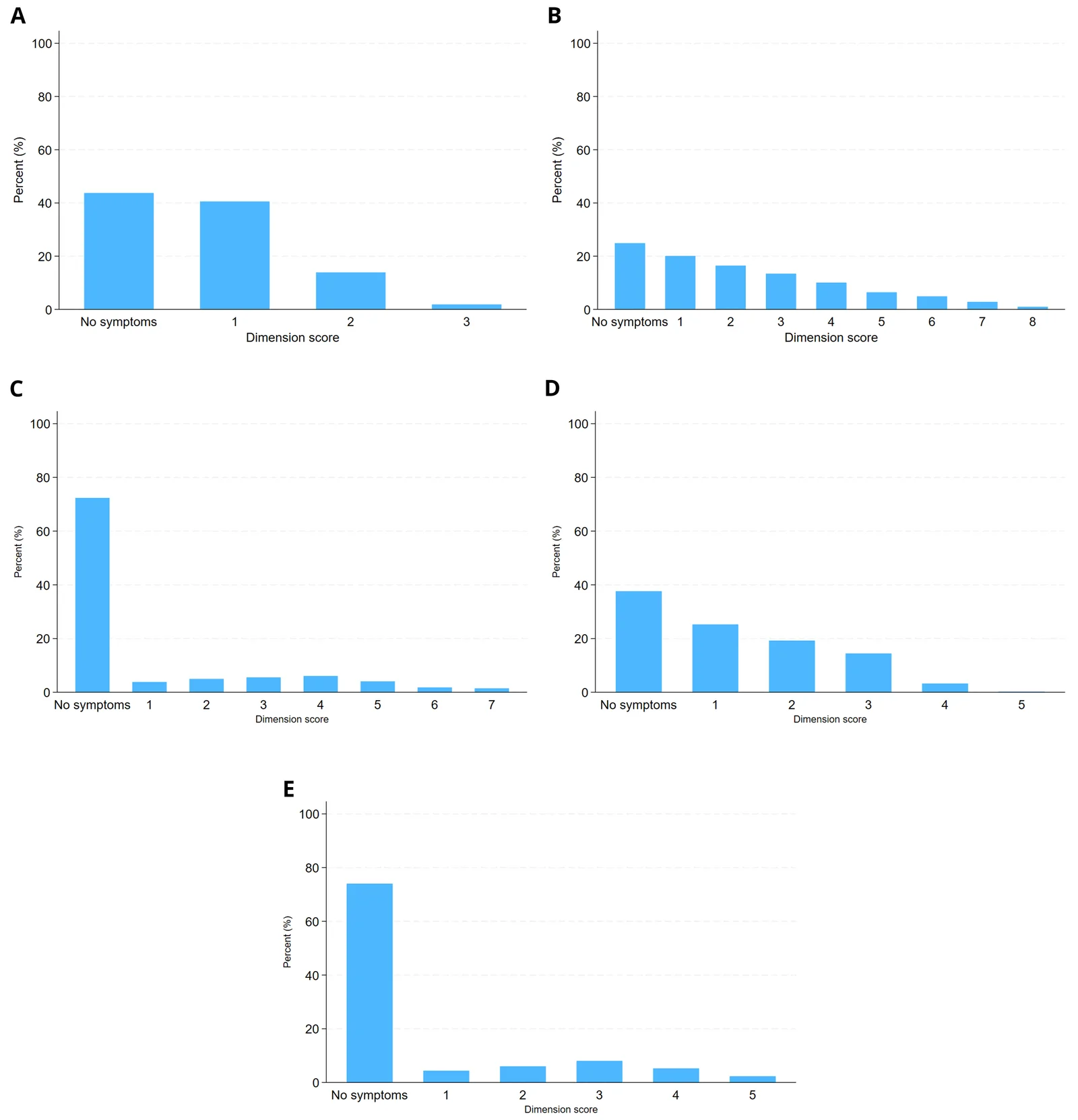
Bar charts showing distributions of symptom dimension scores (level 2). ( **A**) Atypical symptom dimension, ( **B**) Somatic symptom dimension, ( **C**) Psychological symptom dimension, ( **D**) Anxiety symptom dimension, ( **E**) Depressive ideas symptom dimension.

The F@24 clinic was conducted between June 2015 and October 2017, when participants were approximately 24 years old. Of 9,997 participants invited to take part, 4,026 attended, and there is data available from approximately 3,967 people at the time of writing to create level 1-4 variables (note that exact numbers may change due to participant withdrawals from the study). Table 2 shows the prevalence of ICD-10 diagnoses for depression and anxiety in the sample. Approximately 11% of the F@24 clinic attendees met the criteria for depression (mild, moderate, or severe). Approximately 10% of respondents met the criteria for GAD. Table 3 shows the total number of responses for each derived variable and the distribution across each ordinal variable, giving the sample size and percentage of respondents. Somatic symptoms were the most common, with 75% of the study population reporting symptoms. Depressive ideas (e.g., self-harm and hopelessness) were the least common, with 26% of the population reporting symptoms. Figure 4 shows the distribution of the Level 2 symptom dimension variables. Table 4– Table 6 show the frequency of each binary and categorical variable from Level 1 to Level 4, giving the sample size and percentage of respondents. Table 7 and Table 8 show level 1 diagnoses and F@24 clinic attendance by sex.

**Table 2.** Prevalence of ICD-10 diagnoses of Depression and Anxiety (L1 variables).

	Total (n)	Diagnosed – n (%)
Depression (any severity)	3964	432 (11)
Moderate to Severe depression	3964	304 (8)
Severe depression	3964	53 (1)
Any anxiety disorder	3941	398 (10)
Generalized anxiety disorder	3955	386 (10)

**Table 3.** Score frequencies and distributions for L2 symptom dimension variables ^2^.

	Score distributions, n (%)									
	Total (n)	0	1	2	3	4	5	6	7	8+ ^3^
Atypical	3927	1717 (44)	1592 (41)	545 (14)	73 (2)	-	-	-	-	-
Somatic	3832	953 (25)	769 (20)	630 (16)	514 (13)	385 (10)	246 (6)	188 (5)	109 (3)	38 (1)
Psychological	3932	2845 (72)	150 (4)	195 (5)	217 (6)	239 (6)	(4)	70 (2)	57 (1)	-
Anxiety	3835	1443 (38)	968 (25)	737 (19)	554 (14)	125 (3)	8 (<1)	-	-	-
Depressive ideas	3942	2918 (74)	173 (4)	236 (6)	317 (8)	207 (5)	91 (2)	-	-	-

**Table 4a.** Score frequencies and distributions for L3 symptom variables – ordinal.

	Score distributions, n (%)					
	Total (n)	0	1	2	3(+) ^4^	4
Fatigue	3972	1990 (50)	290 (7)	399 (10)	576 (15)	717 (18)
Concentration and forgetfulness	3930	3070 (78)	534 (14)	230 (6)	96 (2)	-
Irritability	3946	2591 (66)	447 (11)	546 (14)	266 (7)	96 (2)
Worry	3934	1988 (51)	771 (20)	473 (12)	347 (9)	355 (9)
Anxiety	3934	2748 (70)	504 (13)	272 (7)	223 (6)	187 (5)
Phobias	3946	3787 (96)	81 (2)	68 (2)	10 (<1)	-
Panic	3934	3798 (97)	6 (<1)	42 (1)	65 (2)	23 (1)
Depression	3951	3170 (80)	361 (9)	251 (6)	159 (4)	10 (<1)
Aches and pains	3971	2578 (65)	439 (11)	412 (10)	317 (8)	225 (6)
Sleep problems	3924	2532 (65)	372 (9)	638 (16)	285 (7)	97 (2)
Appetite/weight change	3943	2752 (70)	419 (11)	516 (13)	256 (6)	-

**Table 4b.** Score frequencies and distributions for L3 symptom variables – discrete.

	Total (n)	Category	N (%)
Psychomotor change	3955	no change	3242 (82)
		either agitation or slowing	448 (11)
		both agitation and slowing	265 (7)
Hopelessness	3957	no	3150 (80)
		yes	807 (20)
Low self esteem	3958	no	3116 (79)
		yes	842 (21)
Decreased libido	3958	no	3532 (89)
		yes	416 (11)
Self-blame	3956	never	2971 (75)
		only when at fault	250 (6)
		sometimes	492 (12)
		often	243 (6)
Diurnal variation in mood	3956	no variation	3176 (80)
		worse in morning	157 (4)
		worse in afternoon/evening	286 (7)
		sometimes worse AM, sometimes PM	337 (9)
Self-harm thoughts	3958	no	3806 (96)
		yes	152 (4)
Passive suicidal thoughts	3957	no	3584 (91)
		yes	373 (9)
Thoughts of suicide method	3960	no	3830 (97)
		yes	130 (3)

**Table 5a.** Score frequencies and distributions for L4 directionality variables – ordinal.

Score distributions, n (%)							
	Total (N)	0	1	2	3	4	5
Sleeping less	3932	2607 (66)	276 (7)	475 (12)	359 (9)	161 (4)	54 (1)
Sleeping more	3953	3702 (94)	33 (1)	120 (3)	71 (2)	27 (1)	-

**Table 5b.** Score frequencies and distributions for L4 directionality variables – discrete.

	Total (n)	Category	n (%)
Psychomotor agitation	3952	no	3493 (88)
		yes	459 (12)
Psychomotor slowing	3956	no	3437 (87)
		yes	519 (13)
Appetite increase/weight gain	3946	none	3248 (82)
		appetite increase only	242 (6)
		weight gain (< 7 pounds)	309 (8)
		weight gain (≥7 pounds)	147 (4)
Appetite decrease/weight loss	3963	none	3381 (85)
		appetite decrease only	246 (6)
		weight loss (< 7 pounds)	223 (6)
		weight loss (≥7 pounds)	113 (3)

**Table 6.** Level 1 diagnoses by sex.

Diagnosis	Male, n (%)	Female, n (%)	Total, n (%)	χ ^2^	P-value
Depression (any)	100 (23)	331 (77)	431 (100)	41.04	<0.001
Moderate to severe depression	69 (23)	235 (77)	304 (100)	29.98	<0.001
Severe depression	12 (23)	41 (77)	53 (100)	4.93	0.03
Anxiety (any)	99 (25)	299 (75)	398 (100)	29.02	<0.001
Generalized anxiety disorder	96 (25)	290 (75)	386 (100)	28.25	<0.001
Specific/isolated phobia	<5 (<1) ^5^	7 (100)	7 (100)	4.16	0.04
Panic disorder	7 (19)	29 (81)	36 (100)	4.95	0.03

**Table 7.** Prevalence of depression in ALSPAC by sex.

	Male, n(%)	Female, n(%)	Total
Not depressed	1377 (93)	2153 (87)	3530
Depressed	100 (7)	331 (13)	431
Total	1477	2484	3961

**Table 8.** ‘Focus at 24+’ Clinic attendance by sex.

Sex	Attended F@24 clinic, n (%)	Did not attend F@24 clinic, n (%)	*χ* ^2^	*P*
Male	1502 (20)	6162 (80)	410.6	<0.001
Female	2513 (34)	4824 (66)		

## Considerations for the data

### Differences between described data and the original CIS-R

The CIS-R is a flexible tool that can be adapted to cover a broader or narrower range of clinical symptoms or disorders, depending on the need/scenario. A shortened version of the CIS-R was used at age 24 years in ALSPAC, which excluded certain conditions (OCD and PTSD). Below, we describe the differences between the CIS-R used in ALSPAC at the age of 24 compared to the original CIS-R. We also described some minor differences in how we used the raw data to derive symptom scores described in this data note. However, as far as possible, we aimed to follow the original questionnaire structure.

It is also worth noting that the CIS-R variables derived from data at ages 18 and 24 in the ALSPAC resource were derived using similar coding protocols, but the derivation of variables was not identical at these two time points. Researchers should consider potential differences when using the CIS-R data from both time points for longitudinal analyses.

The ways in which the data described here differs from the original CIS-R are described below:

- Items measuring ‘compulsions’ and ‘obsessions’ as individual symptoms in the original CIS-R were not described here. These were excluded from the F@24 CIS-R to limit the questionnaire to the evaluation of depression and generalized anxiety disorder, panic, phobia, and social phobia.
- Items regarding ‘ *worry about physical health’* included in the original CIS-R were not included because they also fell outside the scope of the anxiety disorders evaluated at this time point, as described above.
- ‘Somatic symptoms’ described in the original CIS-R are referred to here as ‘ *aches and pains’*. This is due to the original questions referring to the presence of aches/pains/discomfort exclusively when assessing this, whereas clinical definitions of somatic symptoms in the context of depression are broader and additionally include sleep disturbance, appetite disturbance, and fatigue/lack of energy (as characterized by DSM-IV criteria ^62^). Questions referring to aches, pain, and discomfort are available in this data note as individual symptoms and together have been used to construct the ‘somatic symptom dimension.
- We also described items related to self-harm and suicidality, whereas the original CIS-R measures the presence of suicidal cognitions only.

We computed the depressive ideas dimension in line with the CIS-R interview document. However, we have characterised “depressive ideas” as a dimension (level 2) rather than a symptom score, whereas the other CIS-R interview sections are level 3 symptom scores in this data note. This is because the questions in the interview section for depressive ideas each relate to a different cognition (e.g., hopelessness, suicidality), each of which is dichotomized and summed to make the ideation score. This method of computing the score is similar to our method for computing other “dimensions”. The depressive ideas section of the CIS-R interview does not ask questions about the frequency or duration of these cognitions. In comparison, the other sections in the CIS-R are a series of questions related to one symptom (e.g., fatigue or sleep), and the individual will be asked a binary question about whether they have experienced the symptom, followed by questions about frequency and duration.

### Collinearity between some variables

An additional consideration is the overlap between variables. In particular, as mentioned above, Level 2 dimensions are not mutually exclusive from one another (e.g., the atypical and somatic dimensions have overlapping symptoms, similar to depressive ideas and depression dimensions). Furthermore, the individual symptoms (Level 3) described here (e.g., self-blame) are components of the Level 2 dimension variables (e.g., depressive ideas). Therefore, users should be cautious about collinearity in their analyses when using Level 2 dimensions along with Level 3 variables.

### Prevalence of CIS-R measured depression

The CIS-R detects depression cases that are not present in electronic primary care data ^43^, which could be due to the study design (ALSPAC is a population-based cohort study, whereas primary care data can only evaluate service users). The prevalence of CMDs in the ALSPAC cohort reported here was also higher than that in the general population APMS survey from 2014, showing a 6.6% prevalence of GAD and 3.8% prevalence of depressive episodes, with 18.9% of the population having any CMD ^50^. As the APMS is a random probability face-to-face survey ^50^, measurement is influenced by factors such as eligible participants being at home at the time of being approached by the data collection staff. One benefit of population studies such as ALSPAC and APMS is that they are likely to have a better chance of detecting cases within the population that may be missed in primary care. This may be due to a variety of factors related to healthcare access and/or utilisation ^63^. 

### Missing data

Due to the longitudinal nature of the study and decreasing rates of clinic attendance over time, 74% of the overall original (n=15,658) ALSPAC birth cohort sample did not have CIS-R data at age 24. Previous studies have tested whether data are missing at random (MAR) in this sample and found that females and participants whose mothers have higher levels of educational attainment are more likely to be retained in the study ^64^. This means that there is a lower likelihood of data being missing completely at random (MCAR) in this sample; therefore, inferences made from any subsequent analyses should be considered.

## Ethics policy

Ethical approval for the study was obtained from the ALSPAC Ethics and Law Committee and local research ethics committee. Specific ethical approval for the ALSPAC 'Focus at 24+' clinic was granted by the National Research Ethics Service Committee South West – Frenchay: 14/SW/1173 ALSPAC Focus at 24+ (24th February 2015, confirmed 20th March 2015). Further details of the approvals obtained are available on the study website ( http://www.bristol.ac.uk/alspac/researchers/research-ethics/).

## Consent

Informed written consent for the use of data collected via questionnaires and clinics was obtained from participants following the recommendations of the ALSPAC Ethics and Law Committee.

## Data Availability

The ALSPAC data are accessible through a system of managed open access. The steps below highlight how to apply to access ALSPAC data. 1. Please read the ALSPAC access policy which describes the process of accessing the data in detail, and outlines the costs associated with doing so. 2. You may also find it useful to browse the fully searchable research proposals database, which lists all research projects that have been approved since April 2011. 3. Please submit your research proposal for consideration by the ALSPAC Executive Committee. You will receive a response within 10 working days to advise you on whether your proposal has been approved. If you have any questions about accessing the data, please email: alspac-data@bristol.ac.uk. Data sharing statement: The data used for this submission will be made available upon request to the ALSPAC executive committee ( alspac-exec@bristol.ac.uk). The ALSPAC data management plan (available here: http://www.bristol.ac.uk/alspac/researchers/data-access/) describes in detail the policy regarding data sharing through a system of managed open access. ***Supplementary material*** Supplementary materials are available on the Open Science Framework: “Assessment of common mental disorders in the Avon Longitudinal Study of Parents and Children (ALSPAC) birth cohort at age 24 years using the Clinical Interview Schedule-Revised (CIS-R)” project repository, accessed here: https://doi.org/10.17605/OSF.IO/9SQ7R ^65^. This project repository contains the following extended data: CIS-R F@24_syntax.do (Stata code used to create CIS-R variables, Stata file). Supplementary data tables.pdf (Tables describing the CIS-R variable derivation process in more detail): Supplementary Table 1: ICD-10 diagnosis variable derivation process Supplementary Table 2: Coding framework and formulas for all CIS-R variables Supplementary Table 3: Core CIS-R ALSPAC variables at age 24 and details of usage for deriving the described variables. Data are available under the terms of the Creative Commons Attribution 4.0 International license (CC-BY 4.0).
