# Supplementary material for: Assessment of common mental disorders in the Avon Longitudinal Study of Parents and Children (ALSPAC) birth cohort at age 24 years using the Clinical Interview Schedule-Revised (CIS-R)
Source: Wellcome Open Res. 2026 Apr 24;10:680. Originally published 2025 Dec 22. [Version 2] doi: 10.12688/wellcomeopenres.23933.2 (PMC13554738; doi:10.12688/wellcomeopenres.23933.2)
Supplement: Peer review history

# Reviewer report: Reviewer response for version 2

**DOI:** 10.21956/wellcomeopenres.29180.r156810

**Published:** 2026-07-20

**Authors:** Madhavaram Thomas Kishore

**Affiliations:** 1 National Institute Of Mental Health & Neuro Sciences, Bengaluru, Karnataka, India

I am thankful to the editorial team for inviting me to review the above research study. As the authors have correctly identified, depression and anxiety are highly prevalent and often manifest first between childhood and early adulthood. Without early identification and intervention, these conditions can have adverse outcomes throughout lifespan. Therefore, studies like this are welcome.

 The study is based on the Avon Longitudinal Study of Parents and Children (ALSPAC) birth cohort and aims to describe the characteristics of the Clinical Interview Schedule-Revised (CIS-R) data collected from ALSPAC participants aged 24 years. CIS-R provides information on individual symptoms, symptom dimensions, and ICD-10 diagnoses for depression and presence of a range of other CMDs. If the findings of this study are contrasted with the data of the earlier time points, it has a huge potential to provide insights into risk and prognostic factors of depression and anxiety from a longitudinal perspective.

 The research design and the methods adopted to meet the aim of the study are apt, feasible, and replicable. However, I encourage the authors to consider the following points before generalizing the findings:

 1.       The scientific rationale for excluding OCD and PTSD is not strong.

 2.       Since the study findings are based on CIS-R data, fidelity of the assessment process needs to be highlighted. Especially, the measures taken to address assessment variability across the participants.

 3.       The authors have mentioned that the CIS-R questionnaire sections are gated whereby all individuals are asked “mandatory” questions for each section. Those who respond “no” to these questions are not asked further questions in the section. While this process is simple and useful for true positives and true negatives, it is not clear how reliable it is in identifying the false negative cases of depression. Therefore, I encourage the authors to present any studies that have examined the predictive validity of the CIS-R, especially the negative predictive validity, and how satisfactory the findings are. This will help the readers understand the scope and limitation of the ‘gated approach’ of CIS-R to identify common mental disorders like depression and anxiety.

 4.       As the authors have correctly identified, the symptom dimensions are not mutually exclusive, it can lead to collinearity, affecting the reliability of the *dimension scores (level 2*). In this regard, I suggest that the authors clarify whether collinearity was an issue in this study, especially in understanding the main effects (see Ref Kim J. H.). 

 Best wishes,

 Reviewer

Are sufficient details of methods and materials provided to allow replication by others?

Yes

Is the rationale for creating the dataset(s) clearly described?

Yes

Are the datasets clearly presented in a useable and accessible format?

Yes

Are the protocols appropriate and is the work technically sound?

Yes

Reviewer Expertise:

I have expertise in child and adolescent mental health from a clinical psychology perspective. I am well versed with cohort management, and handling large data sets.

I confirm that I have read this submission and believe that I have an appropriate level of expertise to confirm that it is of an acceptable scientific standard.

## References

[1] : Multicollinearity and misleading statistical results. *Korean Journal of Anesthesiology* .2019;72(6) : 10.4097/kja.19087 558-569 10.4097/kja.19087 PMC690042531304696

# Reviewer report: Reviewer response for version 1

**DOI:** 10.21956/wellcomeopenres.26403.r147434

**Published:** 2026-03-06

**Authors:** Cristiane Duarte, Tenneill Murran

**Affiliations:** 1 Psychiatry, New York State Psychiatric Institute, New York, New York, USA; 2 Columbia University, New York, New York, USA

The Data Note reports on the use of the Clinical Interview Schedule-Revised (CIS-R) as part of the ALSPAC study at age 24, with a general focus on reporting on ‘individual symptom dimensions of depression and anxiety required to understand the complex and heterogeneous presentation of CMDs’. Specifically, the CIS-R version is used to evaluate depression and generalized anxiety disorder, panic, agoraphobia, specific phobia, and social phobia. Information about the data is summarized as frequency of disorders (depression and anxiety), symptom domain scores (five) and individual symptoms. Through detailed description of the CIS-R in ALSPAC, the authors aimed to improve standardization and reproducibility of results. The Data Note describes in detail the CIS-R data collection and formatting in the ALSPAC for participants at age 24 years, together with specific demographic information.

 The Data Note is certainly a valuable resource and overall is likely to achieve its objective of improving reliability and integrity in the analysis of the ALSPAC data. We list below the areas for improvement we identified in the data report.

- The Abstract and the text refer to the CIS-R being ‘validated’. Technically, no instrument is ever ‘validated’, but some have substantial information supporting its validity in relation to a specific gold standard and in a specific (or across several) context(s).
- More information supporting its choice of the CIS-R for the ALSPAC study would be important. In the introduction, the paper utilized to support the ‘validity’ of the CIS-R (Lewis et al, 2001) is, in reality, a paper describing a reliability study of the use of the CIS-R by lay and clinically trained interviewers.  Additional information about CIS-R psychometric properties is provided in the Methods section, when describing the CIS-R, however, the only study supporting good sensitivity and specificity of the measure is in an older sample. Information supporting the validity in other age groups would be helpful, or if nonexistent, that should be noted.
- The Introduction should also include the rationale and references supporting the choice of symptom dimension and the specific granular constructs evaluated (e.g. fatigue, concentration, forgetfulness, and hopelessness).
-  It would be helpful to clarify what ‘face-to-face assessment clinics’ mean.
- The Data Note states, ‘The CIS-R also includes ‘a preliminary section on physical health symptoms that is not described here’. It is unclear if the answers to this section have any implication to which of the 14 modules are asked or skipped. If no implications, we suggest this is clearly stated.
- Table 1 and Figure 2 are both very helpful visual displays how the study data was categorized.
- It is unclear to us why the authors chose to refer to the four types of analysis conducted as ‘Levels’. The word ‘level’ implies a ‘scaled’ relationship between the variables created.
- Our main concern, however, is the meaning of some of the continuous scores proposed (Levels 2, 3 and total symptom score) given the ‘gated’ nature of the ALSPAC CIS-R. This means that one is assuming that a ‘no’ to a gate question would be the same as a ‘no’ to all subsequent questions related to that same domain, if the answer to the gate question was ‘yes’, and all questions had been asked. This is a valid and widely used approach for diagnostic interviews; however, generating scaled scores out of information obtained using the ‘gated’ strategy is a questionable practice. We consider, therefore, that what is being labeled as Levels 2 -4 and the total scores (reported in Tables 3-5 and Figure 4) are reflecting questionable information. We are concerned that the inclusion of these variables in a publicly available resource will disseminate disinformation. To exemplify our point: the interpretation of the last row in Table 3 as indicative that  4% of the sample would have a 1 score for depressive ideas is likely incorrect, as if all among the  2,918 score zero participants who did not get asked all the questions had been asked all question, there would like be many more indicating having at least one symptom. In this case, the percentages would be, as expected, with higher percentages for the lowest scores and lower percentages for the highest scores (the current percentages for scores 1 through 5 are 4%, 6%, 8%, 5%, and 2%, respectively). If researchers are seeking a valid continuous measure of some of the dimensions covered in the Data Report, they should be encouraged to use, for example, the MFQ. Finally, the authors included as one of the aims of the paper to encourage the standardization of the use of CIS-R variables. “Historically, ALSPAC data users have combined CIS-R items in different ways for bespoke use; an additional aim of this data note is to encourage the standardization and consistency of data use.” To realize this aim, it would be helpful to describe situations that would be most appropriate for the use of specific variables.

Are sufficient details of methods and materials provided to allow replication by others?

Partly

Is the rationale for creating the dataset(s) clearly described?

Partly

Are the datasets clearly presented in a useable and accessible format?

Yes

Are the protocols appropriate and is the work technically sound?

Partly

Reviewer Expertise:

child psychopathology and epidemiology

We confirm that we have read this submission and believe that we have an appropriate level of expertise to confirm that it is of an acceptable scientific standard, however we have significant reservations, as outlined above.

# Response

**Published:** 2026-04-14

**Authors:** Sophie Fairweather

**Affiliations:** Population Health Sciences, University of Bristol Medical School, Bristol, England, UK

**# COMMENT 1:** The Abstract and the text refer to the CIS-R being ‘validated’. Technically, no instrument is ever ‘validated’, but some have substantial information supporting its validity in relation to a specific gold standard and in a specific (or across several) context(s).   We have updated text in the abstract and introduction. The text now reads as follows: *“The Clinical Interview Schedule-Revised (CIS-R) is an internationally recognised, standardised semi-structured interview tool for assessing CMD symptoms in large community-based samples. There are studies supporting its validity in a range of contexts.” * We have added an additional supporting reference (Das-Munshi et al 2014). The authors assessed the factorial validity of the CIS-R across ethnic minority groups, using data from a nationally representative survey conducted in England in 2000. Respondents comprised people from the five main ethnic minority groups living in Britain (Irish, Indian, Pakistani, Bangladeshi, Black Caribbean), as well as a White British group, aged 16 to 74).  

**# COMMENT 2:** More information supporting its choice of the CIS-R for the ALSPAC study would be important. In the introduction, the paper utilized to support the ‘validity’ of the CIS-R (Lewis et al, 2001) is, in reality, a paper describing a reliability study of the use of the CIS-R by lay and clinically trained interviewers.  Additional information about CIS-R psychometric properties is provided in the Methods section, when describing the CIS-R, however, the only study supporting good sensitivity and specificity of the measure is in an older sample. Information supporting the validity in other age groups would be helpful, or if non-existent, that should be noted.   The CIS-R was developed in the early 1990s and has been widely used since. The CIS-R is an advancement upon existing questionnaires by creating a semi-structured interview that mimics real-world clinical assessment by asking detailed, qualifying questions. This improves its validity against clinical diagnoses vs other diagnostic and screening questionnaires. The use of CIS-R in ALSPAC is well supported by its use in epidemiological studies e.g., in the Adult Psychiatric Morbidity Survey conducted by the UK Office for National Statistics (ONS) which includes adults aged 16 and over living in England. The ONS is the UK's largest independent producer of official statistics and the recognised national statistical institute of the UK. We also direct the reviewer to the following section in the methods: *“The CIS-R has been used to measure the prevalence of treated and untreated psychiatric disorders in the Adult Psychiatric Morbidity Survey (APMS), a large-scale population study conducted every seven years in England and Wales ^50^, and in many randomised controlled trials (RCTs) ^47^. It has been used successfully in different global populations ^51,52 ^and in older populations, with a sensitivity of 75% and specificity of 98% for detecting depressive episodes in people over the age of 60 ^53^.”* We have amended the wording referring to validity in the introduction and added an additional reference (please see response to comment one above).

**# COMMENT 3:** The Introduction should also include the rationale and references supporting the choice of symptom dimension and the specific granular constructs evaluated (e.g. fatigue, concentration, forgetfulness, and hopelessness). Depression and anxiety are phenotypically heterogeneous. The sections of the CIS were originally chosen because they are common symptoms seen in community settings and most were elements of diagnostic criteria. The shortened version here include all the sections required to make diagnoses of depression and anxiety. The chosen symptom domains refer to depression and anxiety symptoms, and symptom dimensions, that are well established in the literature, including ICD-10 diagnostic criteria for depression. These symptoms comprise sections of the original CIS-R questionnaire. We have modified the text under the heading “The Revised Clinical Interview Schedule (CIS-R)”, as follows, adding the text in bold: “… The Revised CIS (CIS-R) was later developed to improve and further standardise assessment when measuring CMDs using the CIS and to increase suitability for administration by non-clinical interviewers in community, occupational, and primary care research. The CIS-R captures presence and severity of core CMD symptoms listed by ICD-10 clinical research criteria (ref: The ICD-10 classification of mental and behavioural disorders: diagnostic criteria for research. 1993. World Health Organization. https://iris.who.int/handle/10665/37108) . It has been shown to be highly specific when detecting CMDs in the population compared to electronic primary care data and can be used to assign ICD-10 diagnostic categories as well as measure individual symptoms.”  

**# COMMENT 4**: It would be helpful to clarify what ‘face-to-face assessment clinics’ mean.   We have amended the wording to read “as part of wider, in-person ALSPAC assessment clinics”. ALSPAC does face to face assessments which are called clinics and postal/online surveys. The data reported here arise from in person/face-to-face “clinic” assessment (as opposed to postal surveys).  

**# COMMENT 5:** The Data Note states, ‘The CIS-R also includes ‘a preliminary section on physical health symptoms that is not described here’. It is unclear if the answers to this section have any implication to which of the 14 modules are asked or skipped. If no implications, we suggest this is clearly stated.   CIS-R is a modular assessment containing different sections. Inclusion or omission of one section (say physical health symptoms or PTSD) does not affect the assessment of other domains (e.g., depression). We have now stated that the answers to this section do not determine whether the rest of the interview sections are completed and do not influence the scores or diagnoses.  

**# COMMENT 6**: Table 1 and Figure 2 are both very helpful visual displays how the study data was categorized.   Thank you. No change needed.  

**# COMMENT 7:** It is unclear to us why the authors chose to refer to the four types of analysis conducted as ‘Levels’. The word ‘level’ implies a ‘scaled’ relationship between the variables created.   The levels refer to hierarchical clustering of symptoms, going from syndrome (diagnosis) to subsyndromal constructs (symptom dimension scores, individual symptom scores). These are clinically meaningful, and well understood by mental health research community.   

**# COMMENT 8:** Our main concern, however, is the meaning of some of the continuous scores proposed (Levels 2, 3 and total symptom score) given the ‘gated’ nature of the ALSPAC CIS-R. This means that one is assuming that a ‘no’ to a gate question would be the same as a ‘no’ to all subsequent questions related to that same domain, if the answer to the gate question was ‘yes’, and all questions had been asked. This is a valid and widely used approach for diagnostic interviews; however, generating scaled scores out of information obtained using the ‘gated’ strategy is a questionable practice. We consider, therefore, that what is being labeled as Levels 2 -4 and the total scores (reported in Tables 3-5 and Figure 4) are reflecting questionable information. We are concerned that the inclusion of these variables in a publicly available resource will disseminate disinformation. To exemplify our point: the interpretation of the last row in Table 3 as indicative that  4% of the sample would have a 1 score for depressive ideas is likely incorrect, as if all among the  2,918 score zero participants who did not get asked all the questions had been asked all question, there would like be many more indicating having at least one symptom. In this case, the percentages would be, as expected, with higher percentages for the lowest scores and lower percentages for the highest scores (the current percentages for scores 1 through 5 are 4%, 6%, 8%, 5%, and 2%, respectively). If researchers are seeking a valid continuous measure of some of the dimensions covered in the Data Report, they should be encouraged to use, for example, the MFQ. Finally, the authors included as one of the aims of the paper to encourage the standardization of the use of CIS-R variables. “Historically, ALSPAC data users have combined CIS-R items in different ways for bespoke use; an additional aim of this data note is to encourage the standardization and consistency of data use.” To realize this aim, it would be helpful to describe situations that would be most appropriate for the use of specific variables.   Thank you for your comment. As the CIS-R is designed as a clinical interview, the use of gating ensures that symptoms are at a clinically relevant level. With the exception of symptoms of depressive ideas, follow up questions relate directly to a gated question, e.g., if a participant responds “no” to not having concentration or memory problems in the past month, it is not possible for them to have had symptoms in the past 2 weeks. However, as referred to by the reviewer, we do agree that the follow up questions relating to depressive ideas do not clearly align to the gated question outside the context of clinical diagnosis. Items under the depressive ideas domain (e.g., hopelessness, low self-esteem, self-blame) follow a gated question as to whether the individual had had a “spell of feeling sad, miserable or depressed in the past week” or “had less or no enjoyment in last week”. This is because the interview is designed to capture depressive ideas in the context of depressed mood, but we agree that these symptoms are probably more prevalent in the population outside of depressed mood. We have expanded the text relating to the depressive ideas dimension under the considerations for use section; we’ve added the following text: “….Additionally, it is important for readers to note that each item that under the depressive ideas symptom domain (e.g., hopelessness, low self-esteem, self-blame) followed a gated question asking whether the individual had had a “spell of feeling sad, miserable or depressed in the past week” or “had less or no enjoyment in last week”. It is possible that these individual symptom scores underestimate the true population prevalence as, for example, someone may have low self-esteem or feelings of self-blame without feeling sad, miserable, depressed or anhedonia. These items should be considered as symptoms in the context of low mood or anhedonia. Continuous measures outside of this may be captured by other measures including the SMFQ.” We have also added a note to supplementary table 3 to make it clear which gating questions (under “Depression (low mood and anhedonia)” section) gate for the depressive ideas items.

# Reviewer report: Reviewer response for version 1

**DOI:** 10.21956/wellcomeopenres.26403.r143936

**Published:** 2026-01-29

**Authors:** Stephanie Ameis

**Affiliations:** 1 University of Toronto Department of Psychiatry, Toronto, Ontario, Canada

The data note presenting the method of administration and characteristics of the CIS-R data collected from ALSPAC participants at age 24years is well written, well presented and provides sufficient detail to allow the research community to use the data for secondary analyses. 

 I have a small number of suggestions/queries for the authors to address/consider.

 1. I did not quite understand the rationale for excluding certain domains in the age 24 wave? For instance, why was the decision made to exclude obsessions and compulsions and PTSD, given these domains are still technically characterized as a common disorder, and especially if these domains were already included in the 18 yo data timepoint?

 2. While it may go beyond the scope of this paper, it would be very useful to report whether the 24 yr data is similar to that acquired at 18 yrs.  That is, do frequencies and distributions appear similar? If there was further data loss such that the 24 yr timepoint differs in demographic characteristics, it would be helpful to understand whether the same patterns that are seen at 24 yrs are seen in the 18 yr data. 

 3. Can initial data be presented indicating the stability of mental health diagnoses/symptom characteristics over time/ bw the 18 and 24 year timepoints.?I think providing some information about stability over time analyzed internally by the ALSPAC group would provide important information to the community and would increase the impact of the paper, as well as increase interest in secondary use of the data.

Are sufficient details of methods and materials provided to allow replication by others?

Yes

Is the rationale for creating the dataset(s) clearly described?

Yes

Are the datasets clearly presented in a useable and accessible format?

Yes

Are the protocols appropriate and is the work technically sound?

Yes

Reviewer Expertise:

Child and youth psychiatry

I confirm that I have read this submission and believe that I have an appropriate level of expertise to confirm that it is of an acceptable scientific standard, however I have significant reservations, as outlined above.

# Response

**Published:** 2026-04-14

**Authors:** Sophie Fairweather

**Affiliations:** Population Health Sciences, University of Bristol Medical School, Bristol, England, UK

**# COMMENT 1.** I did not quite understand the rationale for excluding certain domains in the age 24 wave? For instance, why was the decision made to exclude obsessions and compulsions and PTSD, given these domains are still technically characterized as a common disorder, and especially if these domains were already included in the 18 yo data timepoint? Thank for your comment. The CIS-R data collected from the ALSPAC age 24 clinic (between 2015-2017) omitted questions related to obsessions and compulsions. There is no section for PTSD. A shorter version of CIS-R (dropping sections on OCD) was used to minimise the time required for face to face clinic assessment. During the clinic ALSPAC participants complete a variety of assessments and bio sampling, across several physical, psychiatric, social, educational and other domains. Pressure on time is high, and the cohort administrators make every effort to reduce the testing time where possible.

**# COMMENT 2. **While it may go beyond the scope of this paper, it would be very useful to report whether the 24 yr data is similar to that acquired at 18 yrs.  That is, do frequencies and distributions appear similar? If there was further data loss such that the 24 yr timepoint differs in demographic characteristics, it would be helpful to understand whether the same patterns that are seen at 24 yrs are seen in the 18 yr data.  Thank you for your comment. We have expanded tables 2 and 7 to add frequencies and distributions for depression and anxiety diagnoses at age 18. We have added table 6b. We have added the following text to the materials and methods “Although the aim of this data note is to describe data available at age 24, we have included the overall prevalence of depression and anxiety, and the prevalence split by sex, from the age 18 ALSPAC clinic in Tables 2, 6b and 7 so that readers are able to compare”.

**# COMMENT 3.** Can initial data be presented indicating the stability of mental health diagnoses/symptom characteristics over time/ bw the 18 and 24 year timepoints? I think providing some information about stability over time analyzed internally by the ALSPAC group would provide important information to the community and would increase the impact of the paper, as well as increase interest in secondary use of the data. Thank you for your comment. The aim of this data note is to describe the CIS-R data collected from ALSPAC participants at aged 24 years. As the aim is purely descriptive, analyses of stability/change over time are out of scope. However, we agree these analyses would be valuable. In fact, our research group is investigating as part of a separate ongoing project.
